# Comparison between the Aged Care Facilities Provided by the Long-Term Care Insurance (LTCI) and the Nursing Hospitals of the National Health Insurance (NHI) for Elderly Care in South Korea

**DOI:** 10.3390/healthcare10050779

**Published:** 2022-04-22

**Authors:** Hyeri Shin

**Affiliations:** Department of Gerontology (AgeTech-Service Convergence Major), Graduate School of East-West Medical Science, Kyung Hee University, Yongin 17104, Korea; ltc.shinhyeri@gmail.com; Tel.: +82-31-201-2940

**Keywords:** elderly care, long-term care, needs for elderly, difference in difference

## Abstract

Long-term Care Insurance (LTCI) was created for the elderly, to provide various types of medical and care services, along with the National Health Insurance (NHI). However, the elderly must choose one of these systems, which leads to some of them being unable to receive services and care/medical care based on their needs, because the LTCI only provides limited services, regardless of the needs of the elderly. In order to establish the best solution between the LTCI and NHI, I conducted a utilization effect analysis; using the difference in difference (DID) and difference in difference in difference (DDD) methods with National Health Insurance Services-senior (NHIS-senior) cohort data from 2008 to 2013. The results of the study confirmed that medical expenses are significantly reduced for LTCI users (B = −3.176, *p* ≤ 0.001). Furthermore, when the services meet the older person’s needs, the overall medical expenses are significantly reduced (B = −1.034, *p* = 0.05). These results clearly show that the LTCI is much more suitable for those who need care services. To provide services that more efficiently fulfil their needs, and for better population coverage from the two different systems (the NHI and the LTCI), collaborative work, such as a linkage system, is required.

## 1. Introduction

South Korea implemented a medical insurance system in 1979, and in 2000 the various medical insurance systems were all integrated into the National Health Insurance Corporation (NHIC). Although the NHI is recognized as a stable system, it has a vulnerabilities, in that it lacks the provision of care services for the elderly in their daily lives. Therefore, the NHIC adopted a new system called Long-term Care Insurance (LTCI), in 2008, to provide elderly care services and meet the care needs of the elderly, which were not being met prior to the creation of the system. In effect, the NHIC specifies the care system for older people, according to different sectors: The LTCI focuses on elderly care services, while the NHI covers medical treatment for all ages.

The original goal of creating the LTCI was not only to decrease unnecessary medical expenses for elderly care, but also to allow for its efficient operation. In other words, it reduces social hospitalizations—referring here to a situation in which a patient with a disease that could be treated as an outpatient lives in a hospital for a long period of time—and the NHI and the LTCI systems can function according to their purposes.

Several Korean and international studies have measured medical expenses to investigate the quality and effectiveness of services related to medical treatment and care service for the elderly [1,2,3,4,5,6,7,8,9,10,11,12,13]. To date, many of the South Korean studies and investigations have only focused on the change in the elderly’s medical expenses with the LTCI compared to the NHI [2,12,13]. Kim and Lim [2] analyzed the short-term impact of LTCI, through a regression discontinuity design, to estimate how LTCI impacts informal care and medical expenditures. This result showed that LTCI may help to reduce medical expenses and that it would be more cost-effective for the least able. Lee and Kwak [13] also investigated the use of visiting nursing services from LTCI with medical expenditures, length of hospitalization, and the annual number of ambulatory care visits. This study proved that visiting nursing services were effective services regarding medical expenditures. As you can see from the previous studies [2,13], they only focused on reductions in medical expenses after using the LTCI, or a particular service from among the various services from LTCI. Although medical expenses are an key component in the reduction effect of government funding, it is important to evaluate the system adequacy, such that medical expenditure is still reduced when the elderly patient needs the appropriate services. The majority of previous studies proved that LTCI has resulted in reductions in medical expenses, but they have limitations, in not considering the relationship between the use of LTCI related to the elderly’s needs and medical expenses. Thus, it is necessary to observe those services that specifically meet the elderly’s needs.

This study estimated the effect of the LTCI, by comparing the nursing hospitals using the NHI and aged care facilities using the LTCI in South Korea. A utilization effect analysis was conducted using the difference in difference (DID) and difference in difference in difference (DDD) methods to examine the effect on medical expenses, with consideration of the elderly’s needs: medical treatments at nursing hospitals using the NHI, or care services at aged care facilities using the LTCI system.

## 2. Materials and Methods

### 2.1. Study Population

This study used data from the National Health Insurance Services-senior (NHIS-senior) cohort from 2008 to 2013, from the National Health Insurance Corporation (NHIC): Research Management No. NHS-2016-2-041. The NHIS-senior cohort comprised 10% of South Korean individuals aged 60 years or older in 2002. The database consists of information about individual health status, healthcare utilization, LTCI service utilization, and care type (http://nhiss.nhis.or.kr accessed on 31 December 2021). The study did not employ interviews with staff or older people. Each hospital and aged facility in South Korea entered data of the NHIC. The NHIC stores the data in a central database and then anonymizes it. After random sampling (10%) from all subjects, the NHIC provides the data to researchers. In this study, I examined changes in the reduction in medical expenses of those who received the care services of the LTCI, those who did not receive the care services of the LTCI, and those who received medical services from the NHI, from 2007 to 2013, also considering their medical care needs and care needs.

### 2.2. Dependent Variable: Medical Expenses

To determine changes in medical expenses pre- and post-LTCI introduction, I investigated the total medical expenses between 2007 and 2013 as the dependent variable. Hospitalization fees, medication fees, and injection fees were included, but the cost for dental treatments, oriental medicine, and prescription and non-prescription medicines were excluded.

### 2.3. Independent Variable: Utilization of Services and the Elderly’s Needs

The utilization of the care services provided by the LTCI was the independent variable for this study. Model 1 compared code (1) and comprised the utilization of the care services of the LTCI; home care and aged care facilities users were included, and code (0) refers to a lack of utilization, i.e., non-users of the LTCI. Model 2 compared code (1) and comprised the utilization of the care services of the LTCI at aged care facilities, and code (0) refers to the hospitalized elderly: the utilization of medical treatment at nursing hospitals provided by the NHI.

Moreover, to objectively examine and enhance the accuracy of the elderly’s needs and care, I added a needs variable, as another independent variable based on physicians’ medical opinion letters. Until this study, physicians’ medical opinion letters had not been used as an independent variable in South Korea. The needs were determined by physicians, i.e., whether the elderly patient required the care services of the LTCI or medical treatments from the NHI. Code (1) refers to the need for medical treatment from the NHI, according to a physician’s medical opinion letter. Code (2) refers to the need for combining the care services from the LTCI and medical treatment from the NHI, according to a physician’s medical opinion letter. Code (3) refers to the need for care services from the LTCI, according to a physician’s medical opinion letters. 

### 2.4. DID and DDD Variables: Interaction Variables

The DID variable is the interaction between time and utilization, while the DDD variable is the interaction between time, utilization, and needs. The utilization and needs are the same as the code mentioned above. Additionally, code (1) for time was 2013, and code (2) for time was 2007.

### 2.5. Control Variable

Gender, age, residence (living in urban or rural), disability, household income, living arrangements, the LTCI’s long-term care insurance rates, cancer diagnosis, type of primary caregiver (family or a visiting or live-in caregiver), frequency of receiving care services, and type of insured individual (employee, self-employed, or dependent) were used as control variables.

### 2.6. Statistical Analysis

The purpose of this study was to study the differences in medical expenses based on the elderly patients needs pre- and post-LTCI utilization.

The experimental group was arranged with the utilization of LTCI group, and the control group was arranged with the non-utilization group, with similar tendencies as those of the utilization group. To set up the experimental and control groups with homogeneous propensities, I used the propensity score matching (PSM) method.

The DID and DDD methods were applied to investigate the difference between medical expenses pre- and post-introduction of the LTCI. PSM was used to examine the experimental and control groups, only after the introduction of the LTCI. The PSM method has been used in many previous studies [7,14,15].

Thus, I estimated the PSM after the introduction of the LTCI and set up the experimental group, which was the utilization group, and the control group, which was the non-utilization group. For the matching, variables such as gender, age, region, disability, income, single elderly household, the LTCI’s long-term care insurance rates, cancer diagnosis, type of primary caregiver (family or a visiting or live-in caregiver), frequency of receiving care service, and type of insured individual (employee, self-employed, or dependent) were used as control variables.

Second, I conducted DID and DDD analyses to compare the effects pre- and post-intervention of the services, using a panel regression analysis. Many researchers have used the DID method to examine the effectiveness of the LTCI [5,6] for this reason. On the contrary, the DDD method has not been used to the same extent. The DDD method helps to examine the impact of the system more specifically by comparing additional differences than DID [16].

## 3. Results

The results of the study confirmed that medical expenses are significantly reduced for LTCI users. Furthermore, when the services meet the elderly person’s needs, the overall medical expenses are significantly reduced.

### 3.1. Propensity Score Matching (PSM)

**Figure 1:**
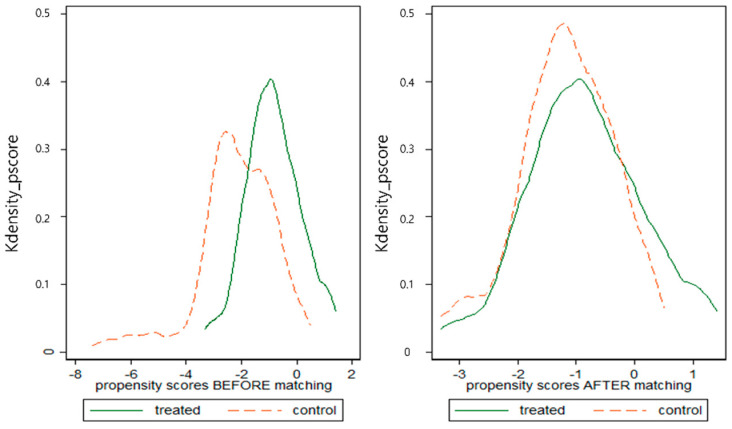
Before and after propensity score matching (PSM).

The homogeneity of the experimental and control groups was established after PSM was confirmed using the graphs in Figure 1. As Figure 1 and Table 1 show, both groups, i.e., the experimental and control groups, had a relatively similar shape after PSM, which means that they had comparable results to those of the PSM.

### 3.2. Panel Regression Analysis

I performed a panel regression analysis for the utilization group of the aged care facilities provided by the LTCI. To compare the utilization group with the LTCI, and the utilization group with the NHI, it was necessary to narrow this down to a utilization group with similar facilities, which was the nursing hospitals for the NHI. The results of the panel regression analysis for the experimental and control groups based on propensity score matching are shown in Table 2.

As a result, I can conclude with three facts: First, the interaction variable between the utilization group and the time variable was significant for medical expenses. In other words, due to the introduction of the LTCI, the medical expenses of the utilization group with the LTCI were reduced compared to those of the non-utilization group for the LTCI. The reduction in medical expenses according to the use of the LTCI was consistent with the results of previous studies [9,10].

Second, the interaction variable of the utilization group, the time variable (2007 and 2013), and the needs (the need for medical treatment, for combining care services and medical treatments, and for care services) were significant for the medical expenses in Model 2, which compared the aged care facilities of the LTCI and the nursing hospitals of the NHI. Thus, when the utilization group with a need for care services used the aged care facilities of the LTCI, their medical expenses decreased after the introduction of the LTCI. This result could be interpreted as a reduction in the medical expenses incurred when the group with the need for care services used the appropriate services. The elderly and their family members who required medical services were more likely to use the medical services appropriately [17,18].

Finally, among the control variables, age, the type of the individual insured with the NHI, income, dementia, cancer diagnosis, and the type of primary caregiver were significant for medical expenses. Similar results were also shown in previous studies [9,10].

## 4. Discussion

This study aimed to evaluate the LTCI and the NHI for elderly care and to determine which system is more efficient and cost-effective for the elderly.

The main results of this study are as follows: First, LTCI utilization was generally significant for medical expenses. This result is consistent with that of a previous study on the LTCI’s effectiveness. Second, it was confirmed that when the elderly used the aged care facilities and required the care services provided by the LTCI, their medical expenses were significantly reduced. I also want to emphasize that the medical expenses for the elderly who needed care services decreased more than for the elderly who needed medical treatment. This result shows that unnecessary medical expenses are reduced when services are appropriate for meeting the elderly’s needs. 

However, the elderly did not use the services according to their own needs, because the two systems are not connected to one another. Full population coverage cannot be achieved on the basis of the elderly’s needs, because the two systems cannot be used together. One system must be chosen at a time. Furthermore, the two systems are not connected, nor can the systems mediate between the LTCI and the NHI for the elderly, which clearly shows that an appropriate linkage plan is required to connect and/or mediate the two systems for the elderly; when services are provided according to the elderly’s needs, the cost of medical expenses goes down and the system operates more effectively. There are, thus, two suggestions for an efficient linkage plan:

First, connecting the two systems using care management should be considered. There are several possible ways to link these two systems, such as integrating the two systems into a single system, connecting only the utilization of services, or having an intermediary system. At present, the two systems, the LTCI and the NHI, are operating in a fairly stable manner, but they do not have cross-functionality, in that many older adults either have to give up their treatments or the services that they are receiving from one of the systems to receive the new treatments or services that they need. Therefore, these two different systems from two different organizations need to develop a solution for linkage, in order to work more effectively and efficiently, and to avoid denying the elderly their rights. The two systems should be slowly combined, and care management should be used to facilitate the utilization of the services of the two systems.

Second, a comprehensive needs assessment should be considered, to meet the elderly’s needs with appropriate services. The care and medical care need level should be determined, guided, and supported, to provide adequate services by a new committee for the LTCI and the NHI. Currently, the Needs Assessment Committee of the LTCI only considers the elderly’s requirements for care services. However, they must reform their considerations and create an integrated needs assessment framework, so that the elderly can receive treatment based on their needs, without complications. 

In this study, there were several implications. First, for the needs variables, this study used physicians’ medical opinion letters, based on the NHIS-senior cohort data from the National Health Insurance Corporation. Previous studies have used the LTCI’s grades instead of the needs factors, but this has the limitation of being an indirect measurement. Therefore, this study was meaningful, in that it utilized the needs variables as evaluated and diagnosed by experts.

Second, this study used NHIS-senior cohort data from the National Health Insurance Corporation as a standardized source of data to increase the accuracy. Previous quantitative studies of the LTCI for the elderly have relied on secondary panel data or surveys, which had a very small target group, ranging from 80 to 150 subjects. On the contrary, this study used data from the NHI, which has the largest utilization group in South Korea and operates the LTCI for the elderly.

In this study, there were a few limitations. First, there were limitations in the physicians’ medical opinion letters: missing contents regarding the elderly and their family’s subjective needs, and a limited quantity of physicians’ medical opinion letters. Second, this study only focused on the reduction in medical expenses, without reflecting on quality of life and psychosocial health.

Despite these limitations, this research proposes a solution for an overall improvement of the LTCI and the NHI. This empirical analysis proved that a linkage system between the LTCI and the NHI could maximize the reduction in medical costs and offer complete services that meet the elderly’s needs.

## Figures and Tables

**Table 1:** General characteristics of the experimental and control groups after PSM.

	Experimental Group	Control Group	*p*-Value
Elderly’s needs	2.02	2.04	
Gender	0.21	0.18	
Age	79.2	79.2	
Type of individual insured with the NHI	1.96	1.96	
Residence	0.41	0.45	
Disability	0.05	0.04	
Household income	5.23	5.19	
Living arrangements	0.055	0.054	
Rates of the LTCI	2.73	2.66	*
Type of primary caregiver	1.27	1.28	
Frequency of receiving care services	2.84	2.79	
Geriatric diseases	0.95	0.960	
Cancer diagnosis	0.028	0.01266	*
*N*	632	174	

Note: * *p* < 0.05.

**Table 2:** Panel regression analysis.

	Utilization of LTCI vs. Non-Utilization		Aged Care Facilities vs. Nursing Hospitals	
	Coef.	S.E.	Coef.	S.E.
Main variable				
Utilization of the LTCI	−0.363 *	0.171	−0.520	0.329
Needs (ref: medical needs)				
Medical care needs	0.079	0.252	0.196	0.334
Care needs	−0.406	0.414	−1.015 *	0.432
Time	0.704 ***	0.041	0.734 ***	0.042
DID (time utilization)	−2.981 ***	0.185	−3.176 ***	0.409
DDD (time utilization needs)	−0.199	0.507	−1.034 *	0.452
Demographics				
Male (ref: female)	0.128	0.120	0.301	0.271
Age	−0.021 **	0.007	−0.029	0.013
Type of individual insured of the NHI (ref: self-employed)				
Employee	−0.012	0.110	−0.280	0.177
Medical aid	0.513	0.152	0.223	0.277
Residence (ref: urban)	−0.024	0.089	0.097	0.156
Disability	0.093	0.228	−0.745 *	0.370
Household income	0.041 **	0.016	0.050	0.029
Living arrangements (ref: living alone)	0.014	0.197	0.221	0.361
Rates of the LTCI	0.152	0.095	0.088	0.153
Geriatric diseases (ref: none)				
Dementia	0.456 ***	0.103	−0.220	0.200
Other diseases	0.383 **	0.123	−0.129	0.225
Cancer diagnosis	0.836 ***	0.248	0.200	0.349
Type of primary caregiver (ref: none)				
Family	−0.328	0.356	−1.153 *	0.456
Other members	−0.231	0.358	−0.834 *	0.425
Frequency of receiving care services	0.038	0.091	−0.0732	0.130
Constant	8.333 ***	0.872	10.34 ***	1.263

Note: * *p* < 0.05, ** *p* < 0.01, *** *p* < 0.001.

## Data Availability

The data were provided by National Health Insurance Sharing Service (http://nhiss.nhis.or.kr accessed on 15 July 2020).
